# Supplementary material for: WASABI: a dynamic iterative framework for gene regulatory network inference
Source: BMC Bioinformatics. 2019 May 2;20:220. doi: 10.1186/s12859-019-2798-1 (PMC6498543; doi:10.1186/s12859-019-2798-1)
Supplement: Supplementary file 1 — Additional file with several tables and figures related to in vitro genes parameters and waves estimation, in silico benchmarking and in vitro GRN candidates fit distance distribution. (PDF 312 kb) [file 12859_2019_2798_MOESM1_ESM.pdf]

# – Supplementary information –

WASABI: A dynamic iterative framework for Gene Regulatory Network inference

Arnaud Bonnaffoux[1,2,3*], Ulysse Herbach[1,2,4], Angélique Richard[1], Anissa Guillemin[1], Sandrine Giraud[1], Pierre-Alexis Gros[3*], Olivier Gandrillon[1,2*]

1 Univ Lyon, ENS de Lyon, Univ Claude Bernard, CNRS UMR 5239, INSERM U1210, Laboratory of Biology and Modelling of the Cell, Lyon, France
2 Inria Team Dracula, Inria Center Grenoble Rhône-Alpes, Lyon, France
3 Cosmotech, Lyon, France
4 Univ Lyon, Université Claude Bernard Lyon 1, CNRS UMR 5208, Institut Camille Jordan, Villeurbanne, France

* arnaud.bonnaffoux@gmail.com
* pierre-alexis.gros@cosmotech.com
* olivier.gandrillon@ens-lyon.fr

Contents

1 *in vitro* genes parameters and waves estimation **2**
1.1 Table and figures of gene parameters and wave times estimation 2
1.2 Protein parameters correlation 2
1.3 Auto-positive feedback coefficient estimation 2

2 *In silico* benchmarking **4**
2.1 Wave time difference in case of auto-positive feedback 4
2.2 *In silico* GRN definition 5
2.3 *In silico* experimental data 6
2.4 *In silico* wave times 7
2.5 *In silico* inference 8
2.5.1 Definition of inference *Quality* 8
2.5.2 Cascade GRN 8
2.5.3 Auto-positive feedback 9
2.5.4 Feedback GRN 10

3 *In vitro* GRN candidates fit distance distribution **11**

1 *in vitro* genes parameters and waves estimation

1.1 Table and figures of gene parameters and wave times estimation

All following files are available at `https://osf.io/gkedt/`. Table *in_vitro_gene_parameters_estimation.csv* provides all genes parameters and wave times. Files *Waves_invitro_1_wave_per_gene.pdf* and *Waves_invitro_2_waves_per_gene.pdf* illustrate wave time estimation respectively in case of one wave per gene or 2 waves per gene. File *Protein_fitting.pdf* illustrates protein fitting for s_1 and d_1 parameter estimation.

1.2 Protein parameters correlation

For the 25 genes that were neither detected in our proteomic data or in literature [1], we estimated parameters with the following rationale: we consider that the non-detection in the proteomic data is due to low protein copy number, lower than 100. Moreover [1] proposed an exponential correlation between s_1 (translation rate) and mean protein level that is confirmed by Fig 1:

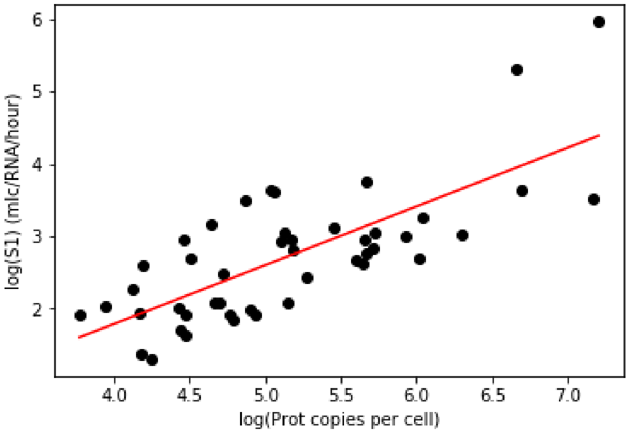

Fig 1. Correlation between s_1 and protein level. Exponential correlation between estimated s_1 and mean protein level. We consider the relation $s_1 = 10^{-1.47} * P^{0.81}$. Linear regression was performed with Python scipy.stats.linregress() function from Scipy package: $r^2 = 0.55$, slope=0.81, intercept=-1.47, p=$2.97 * 10^{-9}$.

1.3 Auto-positive feedback coefficient estimation

Distribution of estimated auto-positive feedback coefficient (Fig 2) from *in vitro* data clearly distinguish 2 groups of genes. One group of 11 genes with a very low coefficient lower than 0.5, and another important group of 79 genes with coefficients greater than 0.5. This result is consistent with assumption of non-autoactivated genes and other influenced by a positive loop. More over, variability of non-null coefficients ranging from 0.5 to 2.25 could carefully be interpreted as presence of strong direct self-activation and weaker positive feedbacks. This last interpretation should be validated by additional work.

Auto-positive feedback coefficients were also estimated from 20 *in silico* GRN embedding autoacivated genes (Fig3). Only genes with auto-positive feedback that are

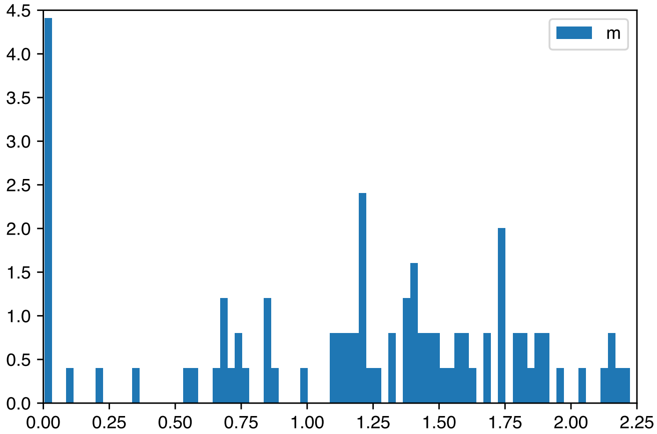

Fig 2. ***in vitro* auto-positive feedback coefficient estimation.** Interaction auto-positive feedback coefficient parameters estimated from time course single cell RNA distribution. This coefficient corresponds to exponent parameter of Hill like interaction function between gene protein against its own promoter parameters. Null value corresponds to absence of positive feedback loop.

activated, and not inhibited, during simulation have an estimated auto-positive feedback coefficient greater than most of other genes. Remarkably, we observe a threshold around 0.45, like for *in vitro* distribution (Fig2). This similitude gives credit to representativeness of our inslico GRN and comfort our choice to set auto-positive feedback detection threshold to 0.45. However, *in vitro* auto-positive feedback coefficients range to 2 while *in silico* ones are limited to 1, suggesting that biological auto-positive feedback are stronger in intensity compare to our model. But this difference has no impact on the definition of auto-positive feedback detection threshold.

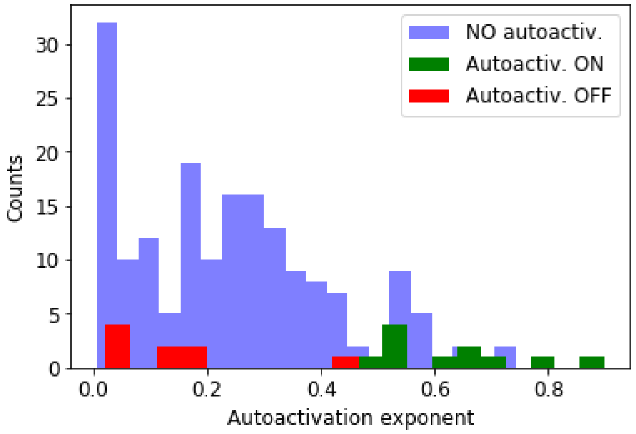

Fig 3. ***in silico* auto-positive feedback coefficient estimation.** Auto-positive feedback coefficients are estimated from *in silico* single cell data. Genes with an auto-positive feedback that are activated during simulation are presented in green, inhibited are presented in red. Genes without auto-positive feedback are presented in blue.

2 *In silico* benchmarking

2.1 Wave time difference in case of auto-positive feedback

To estimate the acceptable range for wave time difference in case of autoactivated target gene, we reuse the 20 *in silico* GRNs previously used for auto-positive feedback coefficient estimation. For each interactions of these 20 *in silico* GRNs we compute the difference between estimated regulated promoter wave time minus its regulator protein wave time. Distribution of promoter/protein wave time difference is given for all interactions considering regulator gene autoactivation status. Distribution of wave times differences is provided in following Fig 4. Acceptable range for wave times difference in case of auto-activation is set to $[-30h, 50h]$.

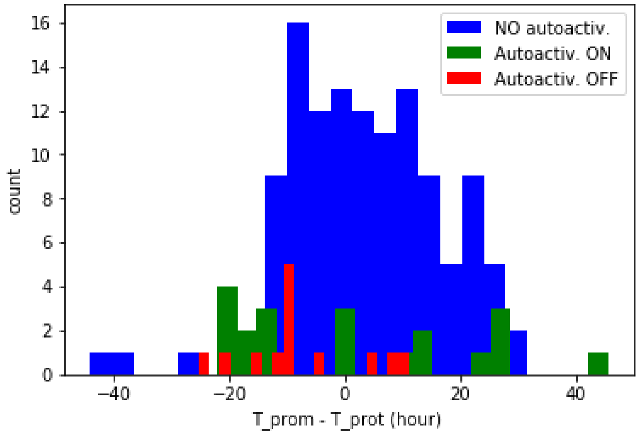

Fig 4. Distribution of wave time difference for auto-positive feedback genes

2.2 *In silico* GRN definition

For *in silico* validation we define 3 GRNs to be inferred which topology is given in Fig 5. Gene's parameters are given in table 1. Interaction parameters are given in table 2.

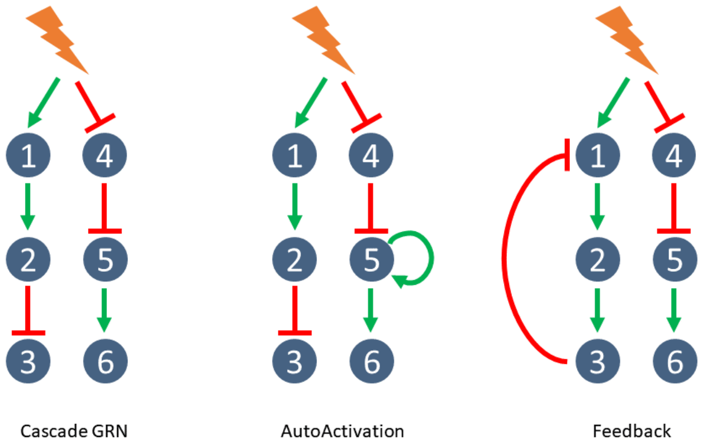

Fig 5. In-silico GRN 3 GRN were designed with different structure pattern to validate WASABI inference

Table 1. *in silico* GRN gene parameters.

parameter	value	unit
d_0	0.173	h^{-1}
s_0	100	h^{-1}
d_1	0.046	h^{-1}
s_1	10000	h^{-1}
$k_{\text{on\_min}}$	0.001	h^{-1}
$k_{\text{off\_min}}$	0.3	h^{-1}
$k_{\text{on\_max}}$	0.1	h^{-1}
$k_{\text{off\_max}}$	2	h^{-1}
dt	0.5	h

Table 2. in silico GRN interaction parameters.

GRN	Regulator	Target	Protein threshold	Efficiency
Cascade	Stim 1	gene 1	0.01	4
	Stim 1	gene 4	0.01	-4
	gene 1	gene 2	6	4
	gene 2	gene 3	5	-4
	gene 4	gene 5	10	-3
	gene 5	gene 6	10	4
Auto-positive feedback	Stim 1	gene 1	0.01	4
	Stim 1	gene 4	0.01	-4
	gene 1	gene 2	6	4
	gene 2	gene 3	2	-4
	gene 4	gene 5	10	-3
	gene 5	gene 6	10	4
	gene 5	gene 5	10	4
Feedback	Stim 1	gene 1	0.01	2
	Stim 1	gene 4	0.01	-4
	gene 1	gene 2	6	4
	gene 2	gene 3	5	4
	gene 4	gene 5	10	-3
	gene 5	gene 6	10	4
	gene 3	gene 1	3.5	-4

2.3 *In silico* experimental data

For the 3 *in silico* network we generate experimental data for time points [0,2,4,8,24,33,48,72,100] hours after continuous step stimulation. Data are available at `https://osf.io/gkedt/`. We simulate 200 cells for single-cell data (RNA counts). The mean of 500 cells gives bulk value for RNA counts and protein concentration (μM).

2.4 *In silico* wave times

For the 3 *in silico* GRNs, wave times for promoter and protein are estimated from simulated bulk data. Wave times are given in hours.

Table 3. ***In silico*** **estimated wave times**:ND = Not Detected

GRN	Gene	$W_{promotor}$	$W_{protein}$
Cascade	4	4.12	12.99
	1	4.26	22.33
	5	15.19	45.50
	2	17.67	44.88
	3	37.88	60.10
	6	40.06	60.72
Auto-positive feedback	1	3.67	16.19
	4	4.07	11.76
	2	18.20	35.02
	3	28.50	33.46
	5	38.40	54.87
	6	52.25	66.69
Feedback	1	-0.90	16.93
	4	-0.71	15.38
	2	12.47	86.84
	5	15.92	40.31
	6	33.00	53.97
	3	37.60	52.75
	1	55.50	ND
	2	65.49	86.84

2.5 *In silico* inference

2.5.1 Definition of inference *Quality*

We note *GRN quality* the inference quality metric that quantifies proportion of true interactions conserved in the candidate network compared to true network. A 100% corresponds to the true GRN. To compute *GRN quality* for a GRN candidate, we first compute for each of its genes a *sub-network quality*, the sub-network corresponds to all paths connecting stimulus to the gene. Then, we compute *GRN quality* as the mean value of all *sub-network qualities*.

sub-network quality is computed for a gene as follow: we estimate the number of intermediaries genes between gene and stimulus in both candidate and true sub-networks. If the numbers of intermediaries is different, *sub-network quality* is null. Else, *sub-network quality* corresponds to the ratio of (i) counts of common interactions between candidate and true sub-networks, and (ii) maximum between candidate and true sub-network sizes (interaction counts).

2.5.2 Cascade GRN

WASABI is run to infer cascade *in silico* network. Interaction consensus matrix Fig 6 is generated for each network candidate with a fit distance lower than 15. Each square in the matrix represents either the absence of any interaction, in dark blue, or the presence of an interaction, the frequency of which is color-coded, between the considered regulator ID (row) and regulated gene ID (column). First row correspond to stimulus interactions. Sign of frequency indicates activation (positive) or inhibition (negative). Green and red circles respectively correspond to true network activations and inhibitions.

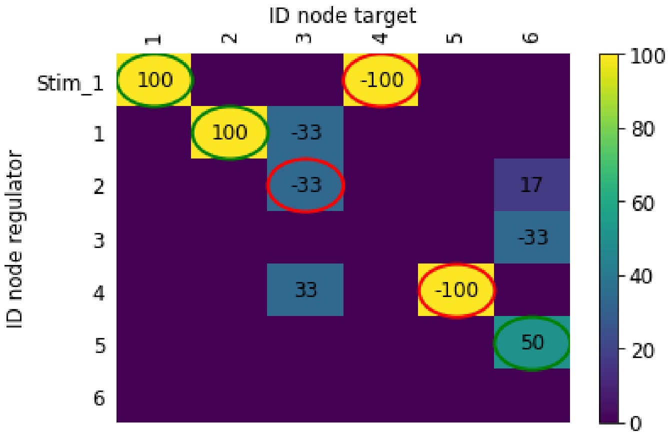

Fig 6. Cascade network consensus interaction matrix.

2.5.3 Auto-positive feedback

Genes auto-positive feedback coefficient are estimated from *in silico* single cell data. According to threshold set to 0.45, only gene 5 of autoactivated network presents an auto-positive feedback. Table 4 gives estimated auto-positive feedback coefficient for all genes of autoactivated network.

Table 4. AutoActivation coefficient estimation.

Gene	Autoactivation coefficient
1	0.19
2	0.21
3	0.067
4	0.14
5	0.65
6	0.28

WASABI is run to infer autoactivated *in silico* network. Fit distance distribution Fig 7 is represented for true GRN (green) and candidates (blue). True GRNs are calibrated by WASABI directed inference while candidates are inferred from non-directed inference. Fit distance represents similitude between candidates generated data and reference experimental data

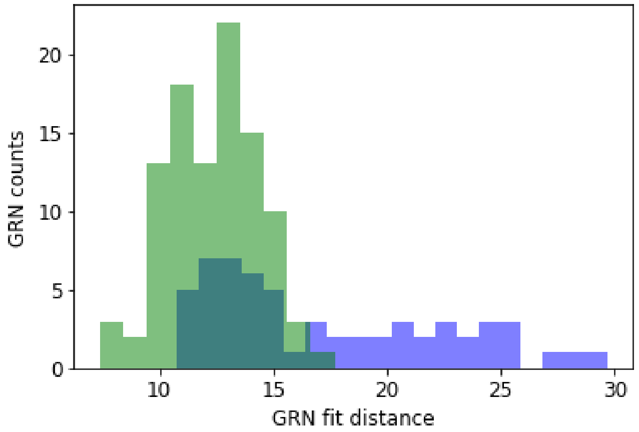

Fig 7. Auto-positive feedback: Fit Distance for true GRN and candidates. Reexpliquer le graph

Interaction consensus matrix Fig 8 is generated for each network candidate with a fit distance lower than 17. See cascade network consensus matrix for figure description.

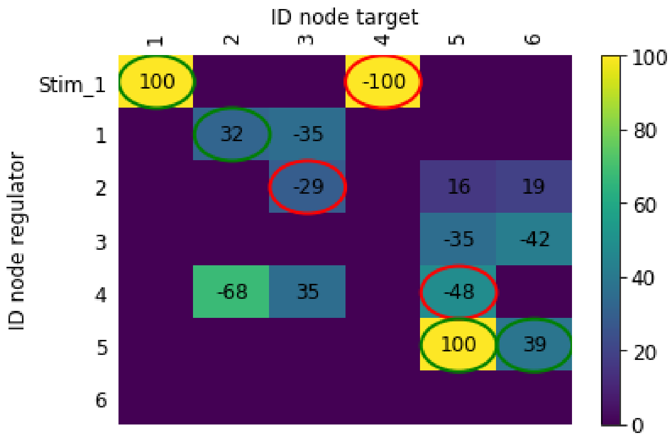

Fig 8. Autoactivated network consensus interaction matrix.

2.5.4 Feedback GRN

WASABI is run to infer negative feedback *in silico* network. Fit distance distribution Fig 9 is represented for true GRN (green) and candidates (blue). True GRNs are calibrated by WASABI directed inference while candidates are inferred from non-directed inference. Fit distance represents similitude between candidates generated data and reference experimental data

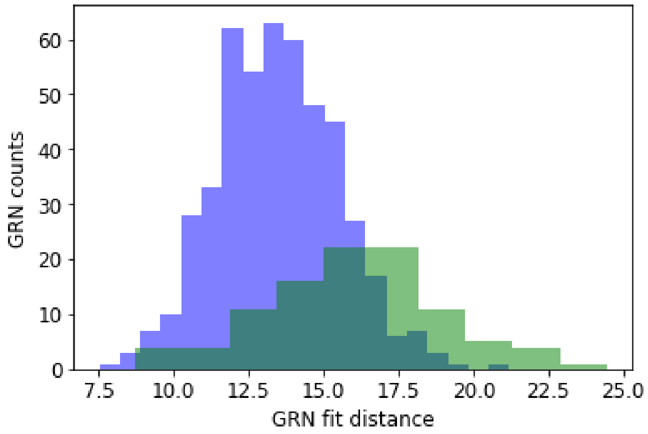

Fig 9. Feedback: Fit Distance for true GRN and candidates.

Interaction consensus matrix Fig 10 is generated for all network candidates. See cascade network consensus matrix for figure description.

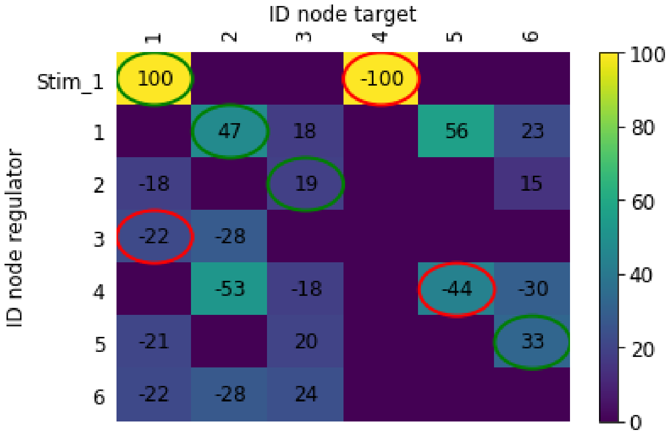

Fig 10. Negative feedback consensus interaction matrix.

3 *In vitro* GRN candidates fit distance distribution

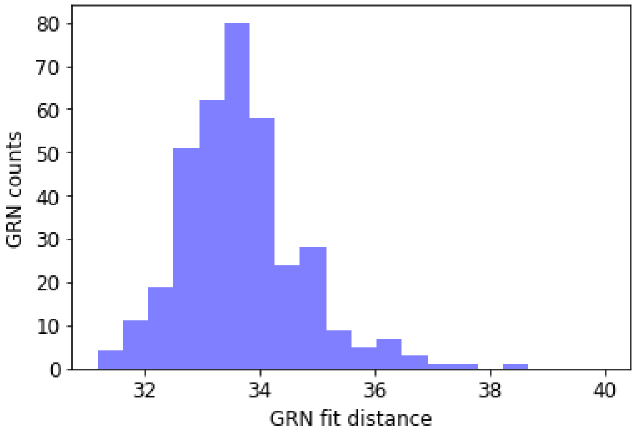

Fig 11. *In vitro* GRN candidates fit distance distribution 364 GRN candidates (excluding outliers) were generated from WASABI application to *in vitro* data.

References

1. Schwanhausser B, Busse D, Li N, Dittmar G, Schuchhardt J, Wolf J, et al. Corrigendum: Global quantification of mammalian gene expression control. Nature. 2013;495(7439):126–127.
